# Altered Timing of Self‐Voice Processing in Parkinson's Disease

**DOI:** 10.1111/ejn.70700

**Published:** 2026-10-14

**Authors:** Francisco Contreras‐Ruston, Antonio Criscuolo, Antonio Callén, David Cucurell, Jordi Navarra, Sonja A. Kotz

**Affiliations:** ^1^ Center for Music in the Brain, Department of Clinical Medicine Aarhus University and the Royal Academy of Music Aarhus/Aalborg Aarhus Denmark; ^2^ Department of Neuropsychology and Psychopharmacology, Faculty of Psychology and Neuroscience Maastricht University Maastricht the Netherlands; ^3^ Department of Cognition, Development and Educational Psychology University of Barcelona Barcelona Spain; ^4^ Speech‐Language Pathology and Audiology Department – Universidad de Valparaíso San Felipe Chile; ^5^ Max Planck Institute for Biological Cybernetics Tübingen Germany; ^6^ Neurology Department Parc Sanitari Sant Joan de Déu Sant Boi de Llobregat Barcelona Spain; ^7^ Institut de Recerca Sant Joan de Déu Esplugues de Llobregat Barcelona Spain; ^8^ Cognition and Brain Plasticity Unit, Neuroscience Program Institut d'Investigació Biomèdica de Bellvitge (IDIBELL) L'Hospitalet de Llobregat Spain; ^9^ Institute of Neurosciences, Universitat de Barcelona Barcelona Spain; ^10^ Department of Neurology Max Planck Institute for Human Cognitive and Brain Sciences Leipzig Germany

**Keywords:** EEG, loudness, Parkinson's disease, sensory feedback, voice

## Abstract

Parkinson's disease (PD) affects voice and speech production, often resulting in reduced vocal intensity and monotonous speech. Recent studies suggest that these changes can be partially explained by altered sensory feedback processing in speech production: individuals with PD (IwPD) may fail to monitor and adapt sensory feedback when producing speech. We investigated PD voice production using a voice playback paradigm and focused on the sensory attenuation typically observed in event‐related responses (ERPs) to the self‐generated voice. When sensory feedback is intact, the P50, N100, and P200 responses to a self‐generated voice are attenuated compared to an externally generated voice. Twenty‐three IwPD and 23 matched healthy controls (HCs) completed the voice playback study with three conditions: self‐generated voice (auditory‐motor [AMC]), externally generated voice (auditory‐only [AOC]), and motor‐only. Linear mixed models assessed group differences in ERP morphology. Groups differed in the N100 latency response: IwPD showed a greater shift in latency between AMC and AOC than healthy controls. Regarding the P200, a significant group‐by‐condition interaction indicated that IwPD, unlike healthy controls, produced larger amplitudes for the self‐generated than the passively listened to voice. Taken together, these findings suggest that the processing of one's own voice in IwPD is altered in terms of temporal precision rather than in the N100 suppression effect per se. This effect was accompanied by an enhanced P200 attentional response. These response patterns in IwPD are indicative of likely progressing difficulties in regulating one's own voice, characteristic of Parkinson's disease.

## Graphical abstract

Using a voice playback paradigm, we found that individuals with Parkinson's disease process their own voice with altered timing rather than reduced suppression: the N100 latency shift between self‐generated and externally presented voice was larger in PD, accompanied by an enhanced P200 response, while P50 and N100 amplitude were preserved.

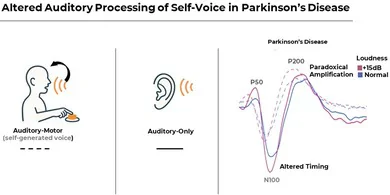

**Abbreviations**
AMC: auditory‐motor condition
AOC: auditory‐only condition
EEG: electroencephalography
ERP: event‐related potential
F0: fundamental frequency
FFR: frequency‐following response
HC: healthy control
IwPD: individual with Parkinson's disease
MOC: motor‐only condition
MoCA: Montreal Cognitive Assessment
PD: Parkinson's disease
SLP: speech‐language pathologist
SPL: sound pressure level
UPDRS: Unified Parkinson's Disease Rating Scale
VAS: visual analog scale

## 1 Introduction

Parkinson's disease (PD) is a progressive neurodegenerative disorder characterized by motor symptoms such as tremor, rigidity, bradykinesia, and postural difficulties (Poewe et al. 2017). Next to motor symptoms, individuals with Parkinson's disease (IwPD) also display nonmotor symptoms, including sensory alterations, that can affect the integration of auditory and proprioceptive feedback. Such compromised sensory feedback processing can affect voice and speech production, manifesting as reduced vocal loudness and imprecise articulation (Ma et al. 2020). In particular, vocal production in IwPD is often marked by hypokinetic dysarthria, which affects voice modulation and articulation and negatively impacts communication and quality of life (Ma et al. 2020).

Voice and speech production involves a sophisticated, multilayered process that relies on the precise interaction between motor planning, motor execution, sensory feedback (i.e., auditory and somatosensory), and adaptation to the acoustic characteristics in the environment (e.g., noise; Blakemore et al. 2000; Knolle et al. 2019; Whitford 2019). Auditory and somatosensory integration engage cortico‐subcortical circuits (including the auditory and motor cortices and the basal ganglia), which play a key role in sensory feedback processing (Nagy et al. 2006; Schwartze and Kotz 2016, 2024; Cardenas et al. 2020). Finding suggests that dopaminergic dysfunction in PD may affect early auditory representations, potentially altering bottom‐up sensory signals that guide vocal motor control (Mollaei et al. 2022). Voice production relies on two complementary processes. First, an adaptive control system integrates motor commands with sensory feedback and environmental cues to regulate vocal loudness; for example, speakers unconsciously increase their loudness in noisy environments by monitoring and adjusting their sensory feedback (Hilger et al. 2022). Second, sensory attenuation allows distinguishing between self‐generated and externally generated sounds, and predictive (forward) models generate an internal estimate of the expected voice signal, which is then compared to the actual auditory input. This comparison leads to the suppression of one's own voice and prevents attentional overload of the auditory system (Knolle et al. 2019). These two processes, real‐time voice adjustment and predictive sensory suppression, support precise voice output and consistent auditory feedback. However, changes in sensory feedback processing can compromise the regulation of vocal loudness and articulatory precision in PD (Huang et al. 2016).

If impaired sensory feedback processing disrupts the modulation of vocal loudness and articulatory precision in IwPD, this might show common voice symptoms, such as hypophonia (Liu et al. 2012; Railo et al. 2020). This symptom results from disruptions in neural circuitry involved in distinguishing between expected and actual sensory feedback. For instance, altered functional connectivity between the basal ganglia and periaqueductal gray regions, which is critical for motor‐sensory integration, may underlie the reduced voice loudness observed in IwPD (Rektorova et al. 2012). Importantly, feedforward projections responsible for expected acoustic features such as loudness and adaptation of actual sensory feedback when expectations are not met, seem both affected in IwPD (Abur et al. 2021). Several studies have indicated that IwPD have difficulties in appropriately anticipating and adapting the acoustic features of their own voices (Liu et al. 2012; Abur et al. 2018). Notably, it was shown that while IwPD can display exaggerated compensatory responses to pitch perturbations, their responses to formant shifts can be markedly reduced. This dissociation suggests that IwPD may differentially weigh acoustic cues in voice motor control, possibly relying more heavily on pitch‐related feedback while underutilizing articulatory‐specific spectral information (e.g., formant structure and the ratio of harmonics to noise) (Mollaei et al. 2016). Given that loudness is also an acoustic feature (i.e., intensity), similar imbalances may extend to its processing, with potential consequences for the regulation of voice output. Clinically, this would manifest as difficulties in accurately perceiving one's own vocal loudness, leading to an overestimation of loudness levels due to impaired sensory feedback processing (Clark et al. 2014). Given that these rapid predictive and corrective voice adaptations typically occur within less than 300 ms (Behroozmand et al. 2016), event‐related potentials (ERPs) offer an effective method to investigate these early stages of sensory feedback processing in PD voice production.

ERPs are sensitive in capturing rapid sensory feedback processing, as they show in millisecond resolution. ERPs such as the P50, N100, and P200 components are indicators of sensory and sensory feedback processing in voice production. For example, the P50 is linked to sensory gating, which is essential for filtering out redundant or irrelevant auditory stimuli (Brinkmann et al. 2021). An increased P50 amplitude in IwPD was interpreted as reduced sensory habituation that is expected in sensory gating (De Groote et al. 2020; Hassin‐Baer et al. 2022).

The N100 has been related to the match/mismatch between expected and actual sensory feedback. The match/mismatch effect shows in a reduced/enhanced N100 response in self‐voice production (Heinks‐Maldonado et al. 2005; Knolle et al. 2019). However, there is currently little to no evidence on the N100 suppression effect in IwPD. Studies that explored the ascribed match/mismatch effect for other effectors in IwPD, such as postural adjustments or adaptation in motor tasks (Olson et al. 2019; Palmisano et al. 2020), argued that similar deficits could be observed in the PD voice and speech production (Huang et al. 2016). These results would explain why brain circuitry that normally optimizes sensory feedback processing and suppresses neural responses to expected sensory input may be affected in IwPD across multiple motor effectors (see Puyjarinet et al. 2019). In healthy individuals, the N100 suppression effect reflects the precision of monitoring expected and actual sensory feedback. In the case of a mismatch, an error that minimizes the suppression effect is elicited (see Knolle et al. 2019). As dopamine might regulate error processing and is depleted in PD (Valdés‐Baizabal et al. 2020), this might lead to less accurate monitoring of errors due to impaired sensory feedback processing in voice production (Huang et al. 2016, 2019). In fact some prior ERP evidence in IwPD reported a reduced N100 suppression effect during vocalization, suggesting a deficit in speech monitoring (Railo et al. 2020). However, such evidence is sparse and has predominantly been explored for pitch modulation, which does not differ between IwPD and HCs (Huang et al. 2016). Thus, the question of sensory feedback processing in the context of voice (loudness) adaptation remains an open question in IwPD.

The last component of interest, the P200 has been related to auditory attention and sensory feedback processing, and the regulation of voice control (Behroozmand et al. 2009; Duggirala et al. 2023). In IwPD, an increased P200 amplitude has been interpreted as enhanced neural responsiveness and a potential compensatory strategy in response to errors in sensory feedback processing (Huang et al. 2016; Railo et al. 2020). Hence, in IwPD, an enhanced P200 response might indicate greater dependence on sensory feedback to regulate voice production (Garrido‐Vásquez et al. 2013; Li et al. 2021). This modulation was also associated with attention to sensory feedback, suggesting that changes in P200 amplitude could reflect a reduction in attentional resources devoted to error processing, implying a reduced ability to detect auditory discrepancies (Behroozmand et al. 2009), or conversely, an increased allocation of attention as a compensatory mechanism in response to sensory error detection (Duggirala et al. 2023).

Despite this evidence, existing ERP data in PD voice production remains limited, especially regarding voice loudness control. Previous studies have predominantly focused on pitch modulation, often yielding inconclusive results regarding sensory feedback deficits in the IwPD (Huang et al. 2016). Thus, the neural correlates underlying the impaired regulation of voice loudness in PD so far remain poorly understood.

In the present study, we recruited IwPD and HC participants to investigate whether PD alters ERP responses (P50, N100, and P200) in a voice playback paradigm. This paradigm complements active vocalization paradigms by isolating sensory feedback from confounding motor activity. We used a well‐established voice playback paradigm inspired by Knolle et al. (2019), and examined ERP morphological changes elicited by self‐generated versus externally generated voice. Based on previous findings, we hypothesized that IwPD should show a diminished sensory attenuation effect and altered ERP amplitudes, reflecting deficits in predictive sensory processing that are critical for accurate voice modulation.

## 2 Materials and Methods

### 2.1 Participants

We recruited a total of 58 participants, including 30 IwPD and 28 HC. IwPD were approached at the Movement Disorders Unit of Neurology at the Parc Sanitari Sant Joan de DéuMHN‐PSSJD in Barcelona. The HCs were recruited from older adult courses at the University of Barcelona and from the family members of IwPD. The experimental protocol included two sessions. In the first session, demographic data (age and sex) were collected from both IwPD and HC, and clinical histories (disease duration and UPDRS‐III speech score) were obtained for IwPD. Cognitive and voice assessments were performed by a speech‐language pathologist (SLP) specializing in the PD voice to characterize vocal status and to rule out vocal disorders unrelated to PD. The second session involved the EEG experiment. All participants provided written informed consent in accordance with the Declaration of Helsinki and regulations of the Human Research Ethics Committee. The study protocol was approved by the Ethics Committee of the Hospital Parc Sanitari Sant Joan de Déu (CEIm: PIC‐27‐23).

During the selection process, a specific exclusion criterion for voice quality was applied. First, an SLP assessed the overall voice quality of all participants (both HC and those with PD), with ratings provided using a visual analog scale (VAS). This allowed to identify and rule out dysphonia, a clinical gold standard in voice therapy (e.g., Zraick et al. 2011; Contreras‐Ruston et al. 2024). These ratings were further complemented by an acoustic analysis to ensure that non‐PD etiologies were excluded. If the voice was found to be dysphonic due to a diagnosed PD‐unrelated voice disorder (e.g., nodules, polyps, cysts, functional dysphonia, or other laryngeal lesions), the individual was excluded from the study. Dysphonia attributable to PD (e.g., hypophonia, tremulous voice, breathy quality, and reduced intensity) was not considered an exclusion criterion, as it presents as a primary disease symptom. Exclusion decisions were based on participants' medical history and the perceptual evaluation by the voice‐specialized SLP. In cases of etiological uncertainty, participants were excluded to ensure that the vocal characteristics analyzed were attributable to PD rather than to other non‐PD pathologies.

Second, hearing was assessed in the IwPD and HC groups. This was further verified using a pure‐tone detection task involving six frequencies (500, 750, 1000, 1500, 2000, and 4000 Hz) presented at five intensity levels. The task started at the highest intensity (50% of the maximum headphone output) and progressively decreased to 40%, 30%, 20%, and 10%. Approximate sound pressure levels ranged from 78/73 dB SPL (low/high frequencies) at 50% to 63/59 dB SPL at 10%. The task was conducted in a soundproof environment using PsychoPy software v2023.3 (Peirce et al. 2019), following a procedure similar to that described by Abur and colleagues (Abur et al. 2018; Abur and Stepp 2020).

Finally, seven PD participants were excluded due to excessive EEG artefacts, a low score on the Montreal Cognitive Assessment (MoCA) for cognitive impairment (≤ 26; Nasreddine et al. 2005), or dropout in the second session. In addition, none of the participants had undergone deep brain stimulation or had any other neurological disorders. The final participant sample included 23 IwPD (14 men and 9 women) aged 52–83 years (SD = 8.33). All IwPD were diagnosed by a neurologist and met the criteria for early and mid‐stage PD (Hoehn and Yahr stages I–III). They were also evaluated using the Movement Disorder Society‐Unified Parkinson's Disease Rating Scale (MDS‐UPDRS) Part III, Item 3.1 (speech) (see Table 1). This item is reported as a standard clinical descriptor of the cohort. On its own, however, this single perceptual rating is a coarse and relatively insensitive index of voice and speech impediment in PD, as objective acoustic analysis can discriminate speech differences that are not perceptually identifiable through the UPDRS speech item (Daoudi et al. 2022). For this reason, vocal status was not characterized by this item alone but by the SLP assessment described above; comparable UPDRS speech scores should therefore not be read as indicating an absence of the subclinical vocal changes that are already present, albeit mild, in early PD (Watts and Zhang 2022). Participants were tested in their self‐reported “ON” state, defined as the period after intake of their regular dopaminergic medication.

**TABLE 1:** Demographic and clinical characteristics of the IwPD.

Individuals	Sex	Age	Disease duration (years)	MoCA	H & Y	UPDRS III (speech)
1	F	74	17	26	II	1
2	M	68	6	26	I	2
3	M	64	3	27	II	1
4	F	57	5	28	I	0
5	F	55	5	27	I	1
6	M	52	4	30	I	2
7	M	62	20	26	I	1
8	F	70	5	28	II	1
9	M	52	1.2	27	II	1
10	M	66	1	27	II	1
11	M	56	2	27	III	1
12	M	74	2	26	I	1
13	M	76	1.6	28	I	1
14	M	66	5	29	III	2
15	F	66	1	27	I	1
16	F	58	2	28	I	0
17	M	73	1.6	27	II	2
18	M	60	1.6	28	II	1
19	M	83	5	26	III	3
20	F	67	7	29	II	0
21	F	57	8	29	II	1
22	F	74	8	29	II	1
23	M	64	11	26	III	1

Abbreviations: F = female; Disease Duration = Years of diagnosis; H & Y = Hoehn and Yahr stage; M = male; MDS‐UPDRS Part III, Item 3.1 (Speech) (0 normal—4 severe); MoCA = Montreal Cognitive Assessment score (range: 0–30; ≥ 26 = normal cognitive level).

For the HC group, 28 participants were recruited from the general population through a recruitment advertisement, of whom five were excluded due to excessive artefacts in EEG signals or dropout, leaving 23 HC (6 men and 17 women) aged between 51 and 70 years (SD = 5.92). In total, 46 participants were included in the study (23 IwPD and 23 HC).

### 2.2 Experimental Design and Procedure

#### 2.2.1 Procedure and Materials

In a first session, we recorded the voices of all the participants in a controlled environment with ambient noise levels below 40 dB. The participants' tasks included producing three sustained/a/vowels, each lasting more than 3 s at their habitual loudness and pitch levels, ascending and descending glissandos, and reading aloud (Patel et al. 2018; Rusz et al. 2021). The recording equipment included a laptop computer running the Audacity software (2023 Audacity Team; https://www.audacityteam.org) and a Karsect HT‐9 microphone with a unidirectional polar pattern connected to an external Focusrite audio interface. The microphone was positioned 5 cm away from the participant's mouth (Patel et al. 2018). The mean baseline SPL of the recorded vowels was 62.4 ± 7.3 dB SPL for IwPD and 62.0 ± 5.1 dB SPL for HC. A Mann–Whitney *U* test indicated no significant difference between groups (*W* = 3025, *p* = 0.264).

In the second session, the most stable of the three vowels produced by each individual participant was selected as the self‐generated stimulus for the EEG experiment. We utilized the playback paradigm described in Knolle et al. (2019), designed to disentangle motor and auditory contributions in self‐voice processing. The paradigm included three blocked experimental conditions (see Figure 1): an auditory‐motor condition (AMC), in which each button press triggered the immediate playback of the participant's recorded vowel for 0.5 s, presented either at its normal loudness or with a + 15 dB increase; an auditory‐only condition (AOC), in which participants passively listened to the same vowel recordings while observing a fixation cross; and a motor‐only condition (MOC), in which participants pressed a button following a visual cue without receiving auditory feedback. Conditions were presented in blocks to separate motor and auditory components and to reduce carry‐over effects. This set‐up has been validated in previous work to probe prediction error and self‐voice monitoring, allowing researchers to characterize how the brain prepares for the sensory consequences of self‐produced sounds and how neural responses change when the produced sound violates this prediction.

**FIGURE 1:**
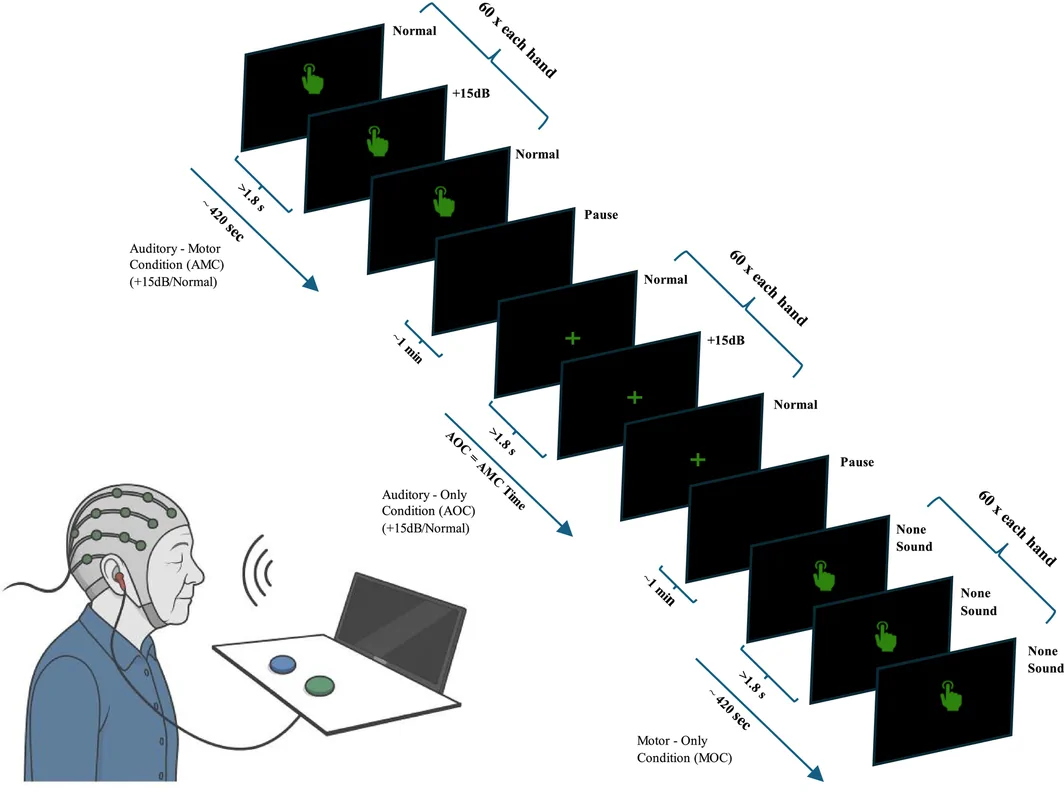
**Playback paradigm**: In the AMC condition, participants pressed a button at intervals > 1.8 s to trigger 420‐ms playback of their own voice (normal/+15 dB) in blocks of ~420 s followed by 1‐min rest; the AOC condition uses identical auditory stimuli and timing but without button presses, participants fixated on a central cross, followed by 1‐min rest; in the MOC condition, participants only press the button at the same cadence (> 1.8 s) in ~420‐s blocks with no auditory stimuli, followed by 1‐min rest. Each condition was repeated 60 times for each hand.

In both AMC and MOC conditions, participants pressed the button approximately every 2.4 s when a fixed visual cue appeared. Button presses within 1.8 s (“too early”) were considered as errors. During the experiment, participants completed a total of 60 button presses with their right hand and 60 with their left hand, distributed in two subblocks (i.e., AMC‐right and AMC‐left), conducted in counterbalanced order across participants. The order in which the stimuli were presented in the AOC was the same as that in the AMC for each participant.

Before starting the experiment, participants underwent preliminary training of the paradigm with the same instructions as in the main experiment to fully understand the task and to ensure that the press between each button occurred at an interval greater than 1.8 s.

### 2.3 EEG Recording

EEG data were recorded using 64 Ag/AgCl actiCAP‐Slim electrodes (BrainAmp, Brain Products) arranged according to the extended 10–20 international system. EEG signals were acquired using two BrainAmp amplifiers, each sampling at 250 Hz. The electrode impedance was maintained below 10 kΩ throughout the recording. The FCz electrode served as an online reference, whereas the Fpz electrode was used as the ground electrode. Data acquisition and storage were performed using Brain Vision Analyzer 2 (Version 2.1.1.2516).

### 2.4 Data Analysis

#### 2.4.1 EEG Preprocessing

The EEG data were preprocessed in MATLAB using the EEGLAB toolbox (Delorme and Makeig 2004). First, line noise (50 Hz) (lineFilter) and its harmonics were removed using a linear‐phase finite impulse response (FIR) filter with a Kaiser window, applied with zero‐phase (forward–backward) filtering and ±2‐Hz stopbands (e.g., 48–52 Hz). This was followed by a band‐pass filter (0.05–35 Hz), also using a linear‐phase FIR design with a Kaiser window, ensuring a zero‐phase response to minimize temporal distortions. Channels with poor signal quality were then automatically identified and removed using (*pop_clean_rawdat*), based on the FlatlineCriterion (≥ 5 s without variation), ChannelCriterion (correlation < 0.8), and Distance = “Euclidean” (detection of atypical channels). Removed channels were subsequently interpolated and the data were re‐referenced to the mastoid electrodes. To ensure precise synchronization between button presses and stimulus presentation, event latencies were corrected by adjusting markers according to the measured delay. A Robotic Key Actuator (RKA) from The Black Box ToolKit (Version 2, Rev. RC2) (Plant and Quinlan 2013) was used to determine and compensate for the mechanical latency of the actuator, allowing for a precise alignment between participant responses and stimulus onset. Relevant triggers for each experimental condition were then identified, and data were segmented into epochs from −2 to +0.5 s relative to these events.

The epoched data were subjected to ICA using (*pop_runica*) in extended mode to decompose the signals into independent components. These components were automatically classified using ICLabel, marking as artefacts those with a probability ≥ 80% for ocular activity and ≥ 60% for muscular activity (*pop_icflag*). Artefacts were removed with (*pop_subcomp*), and the EEG signal was reconstructed from the remaining components to retain genuine brain activity. Finally, an additional cleaning step was applied to remove epochs with amplitudes exceeding ±100 μV using (*pop_eegthresh*) for automatic detection. Additionally, (*pop_jointprob*) was used to exclude epochs with extreme values in joint probability and power distribution, with adaptive thresholds set based on intersubject variability. A final visual inspection ensured the effective removal of residual artefacts and overall quality of the preprocessed data.

#### 2.4.2 Event‐Related Analyses

After preprocessing and artefact rejection, the epochs were averaged separately for each experimental condition to obtain the ERP waveforms. ERP data were processed by averaging electrodes from regions of interest (ROIs), including the frontal (F1, Fz, and F2), frontocentral (FC1, FCz, and FC2), and central (C1, Cz, C2, C3, and C4) areas, following previously established criteria (Rosburg et al. 2008; Knolle et al. 2019; Pinheiro et al. 2019). The following three experimental conditions were included: AMC with two different sound loudness levels (normal and +15 dB), AOC with the same two loudness levels, and MOC.

Baseline correction was applied using an interval of −100 to 0 ms before the stimulus. To isolate the neural responses to sounds from those generated by pressing the button, we subtracted for each participant, the averaged MOC response was subtracted from each AMC single trial. This subtraction was performed separately for each response hand (left‐hand MOC average subtracted from left‐hand AMC trials, and right‐hand MOC average from right‐hand AMC trials), so that motor‐related activity was removed using each participant's own motor baseline before the corrected single‐trial AMC values entered the statistical models. This correction removed the contribution of motor‐related activity associated with movement execution, ensuring that the resulting evoked potentials primarily reflected the auditory response to self‐generated sounds in the AMC, and thus could be compared with those from the AOC. This procedure follows previous sensory suppression playback paradigms (e.g., Knolle et al. 2013b), which allow the evaluation of the impact of sensory feedback processing. For statistical analysis, time windows were defined based on previous studies (Huang et al. 2016; Knolle et al. 2019; Li et al. 2021) and a grand average across all combined conditions (Murray et al. 2008; Luck 2014). The peaks of the P50 (40–75 ms), N100 (90–150 ms), and P200 (190–250 ms) components were extracted, along with their mean amplitudes, variances, peak amplitudes, and latencies. Finally, the number of trials per participant was equalized for each ERP component using random sampling (*randperm*) to ensure a comparable signal‐to‐noise ratio across groups and conditions and to maintain an equivalent amount of data for between‐subject comparisons.

#### 2.4.3 Statistical Analyses

All analyses were conducted in R (Version 4.5.3; R Core Team 2025) within RStudio (Version 2026.01.1). Multilevel models (MLMs) were employed to examine neural response variability at both intra‐ and interindividual levels (Volpert‐Esmond et al. 2021). This approach was appropriate given that models were fitted at the single‐trial level, where each observation represented one ERP trial nested within participants, rather than aggregated as condition‐level means. This nested structure allowed the models to:

1. Relax the assumption of homoscedasticity across trials;
2. Include random intercepts and a random slope for loudness, capturing both baseline differences between participants and individual variability in sensitivity to the loudness manipulation;
3. Appropriately model the dependency structure of trials nested within participants, improving statistical efficiency relative to analyses based on aggregated averages.

Separate models were estimated for each ERP component (P50, N100, P200) and dependent variable (amplitude and latency). For each component, a full model was first fitted including all main effects (group, condition, and loudness) and their two‐way and three‐way interactions, with a random intercept and a random slope for loudness per participant: Mean_Component ~ group × condition × loudness + (1 + loudness participant). The random slope for loudness was included because loudness was the primary within‐subject factor of theoretical interest, allowing modelling of interindividual variability in loudness sensitivity (Barr et al. 2013). Full models were estimated using maximum likelihood (ML) for model comparison.

Model selection followed a backward elimination procedure applied to the fixed‐effects structure (Kuznetsova et al. 2017). Starting from the full model, interaction terms were removed sequentially, from highest to lower order, when they did not reach statistical significance (*α* > 0.05). Main effects were retained regardless of their *p* value, in accordance with the principle of marginality: a main effect was not eliminated if it was involved in a retained higher‐order interaction. The random‐effects structure was held constant across all model comparisons within a given component and dependent variable. Where singular fits were detected, the random‐effects structure was nonetheless retained, given that the random slope for loudness was theoretically motivated rather than data‐driven (Barr et al. 2013). Final models resulting from this procedure were re‐estimated using restricted maximum likelihood (REML) for parameter inference.

Model fit was evaluated using the intraclass correlation coefficient (ICC), quantifying the proportion of total variance attributable to between‐participant differences, and marginal and conditional *R* ^2^ values (Nakagawa and Schielzeth 2013). Marginal *R* ^2^ reflects variance explained by fixed effects alone. Conditional *R* ^2^ reflects variance explained by the full model including random effects. Fit indices were compared between the full and final models to assess whether model simplification altered overall explanatory power.

Significant interactions and main effects retained in the final models were decomposed using estimated marginal means (EMMs) computed with the emmeans package (Lenth 2024). For higher‐order interactions, pairwise contrasts were conditioned on the relevant factor combinations (e.g., loudness within levels of group and condition). All pairwise comparisons were corrected for multiple testing using the Benjamini–Hochberg false discovery rate (FDR) procedure. Effect sizes for pairwise comparisons were expressed as Cohen's *d*, calculated using the residual standard deviation and residual degrees of freedom from each model (Ben‐Shachar et al. 2020). All models were estimated using lme4 (Bates et al. 2015) and lmerTest (Kuznetsova et al. 2017), with *p* values for fixed effects based on Satterthwaite's approximation. Statistical significance was defined as *p* < 0.05.

## 3 Results

### 3.1 Multilevel Analysis

#### 3.1.1 P50

For P50 amplitude analyses, we started with a full model including all main effects and their two‐ and three‐way interactions (group × condition × loudness; see Table S1). In a stepwise fashion, nonsignificant interactions were removed and the model refitted. As none of the interaction terms were significant, the final model retained the three main effects (group, condition, and loudness).

The final model revealed a significant main effect of condition, *F*(1, 6197.4) = 23.99, *p* < 0.001, indicating higher P50 amplitudes in the AOC condition than in the AMC condition (estimate for AMC vs. AOC = −0.88, 95% CI [−1.23, −0.53], *p* < 0.001). No effects of group or loudness survived elimination. The marginal *R* ^2^ was 0.004; the conditional *R* ^2^ could not be estimated (singular fit).

For P50 latency, the full model is reported in Table S2. The final model retained the main effects of group and condition, as no interaction terms reached significance. A significant main effect of condition emerged, *F*(1, 6042) = 53.61, *p* < 0.001, indicating shorter latencies in the AMC condition than in AOC (estimate = −0.0022, 95% CI [−0.0028, −0.0016], *p* < 0.001). The main effect of group approached but did not reach significance, *F*(4.4) = 4.59, *p* = 0.093 (estimate = −0.0007, 95% CI [−0.0013, −0.0001]), with PD trending toward shorter latencies than HC. The marginal *R* ^2^ was 0.009; the conditional *R* ^2^ could not be estimated (singular fit) (see Table S3).

#### 3.1.2 N100

For N100 amplitude, the initial model included all main effects and two‐ and three‐way interactions (see Table S4). The condition × loudness interaction reached significance and was therefore retained; the three‐way interaction with group did not survive elimination.

For N100 amplitude, the effect of condition was significant (*β* = 2.75, 95% CI [2.26, 3.24], *p* < 0.001), as was the condition × loudness interaction (*β* = 1.31, 95% CI [0.62, 2.00], *p* < 0.001); the main effect of loudness approached significance (*p* = 0.093). The marginal *R* ^2^ was 0.054 and the conditional *R* ^2^ was 0.142 (ICC = 0.093) (see Table 2). To decompose this interaction, planned simple‐effects contrasts were conducted within each condition, with FDR correction (see Table S5). Post hoc comparisons revealed that louder stimuli significantly increased N100 negativity in the AOC condition (estimate = −1.09, SE = 0.307, *p* < 0.001), with no significant effect in AMC (estimate = 0.22, SE = 0.307, *p* = 0.472). Correspondingly, the AMC‐AOC suppression effect itself was significantly larger under +15 dB (estimate = 4.06, *p* < 0.001) than under normal loudness (estimate = 2.75, *p* < 0.001).

**TABLE 2:** Effects of group, condition, and loudness on N100 amplitude and latency.

Predictors	Amplitude			Latency		
	Estimates	CI	*p*	Estimates	CI	*p*
Group				0.0009	[−0.0016, 0.0034]	*p* = 0.488
Condition	**2.75**	**[2.26, 3.24]**	***p*** **< 0.001**	**−0.0051**	**[−0.0063, −0.0039]**	**p < 0.001**
Loudness	−1.09	[−1.69, −0.48]	*p* = 0.093	**−0.0024**	**[−0.0032, −0.0015]**	***p*** **< 0.001**
Group × Condition				**−0.0022**	**[−0.0039, −0.0005]**	***p*** **= 0.012**
Condition × Loudness	**1.31**	**[0.62, 2.00]**	***p*** **< 0.001**			
**Random effects**	*σ* ^2^ = 48.59 *τ*₀₀ = 5.20 ICC = 0.093 *R* ^2^ = 0.054/0.142			*σ* ^2^ = 2.983e‐4 *τ*₀₀ = 1.53e‐5 *R* ^2^ = 0.036/n.a.		

*Note:* The table shows fixed‐effect estimates, 95% confidence intervals (CIs), and *p* values from mixed‐effects models predicting N100 amplitude and latency. Significant results are shown in bold. Random effects included variance components (*σ* ^2^ and *τ*₀₀), intraclass correlation (ICC), and marginal/conditional *R* ^2^ values. Group was not retained in the amplitude model (shaded cells); group × condition was retained in the latency model.

For N100 latency, the full model is reported in Table S6. Significant main effects of condition (*β* = −0.005, 95% CI [−0.006, −0.004], *p* < 0.001) and loudness (*β* = −0.002, 95% CI [−0.003, −0.002], *p* < 0.001) were found, indicating shorter latencies in the AMC condition and in response to louder stimuli. A significant group × condition interaction was also retained (*β* = −0.002, 95% CI [−0.004, −0.001], *p* = 0.012), indicating a larger AMC‐related latency shift in IwPD than in HC (see Table 2). The marginal *R* ^2^ was 0.036; the conditional *R* ^2^ could not be estimated (singular fit). Post hoc comparisons confirmed a larger AMC‐AOC latency shift in PD (estimate = −0.0073 s, *p* < 0.001) than in HC (estimate = −0.0051 s, *p* < 0.001) Comparing groups within each condition, direct HC–PD differences were nonsignificant (AOC: estimate = −0.0009 s, *p* = 0.496; AMC: estimate = +0.0013 s, *p* = 0.329), indicating that the interaction reflected a group difference in the magnitude of the condition effect rather than an overall latency difference between groups at either condition level (see Figure 2 and Table 3).

**FIGURE 2:**
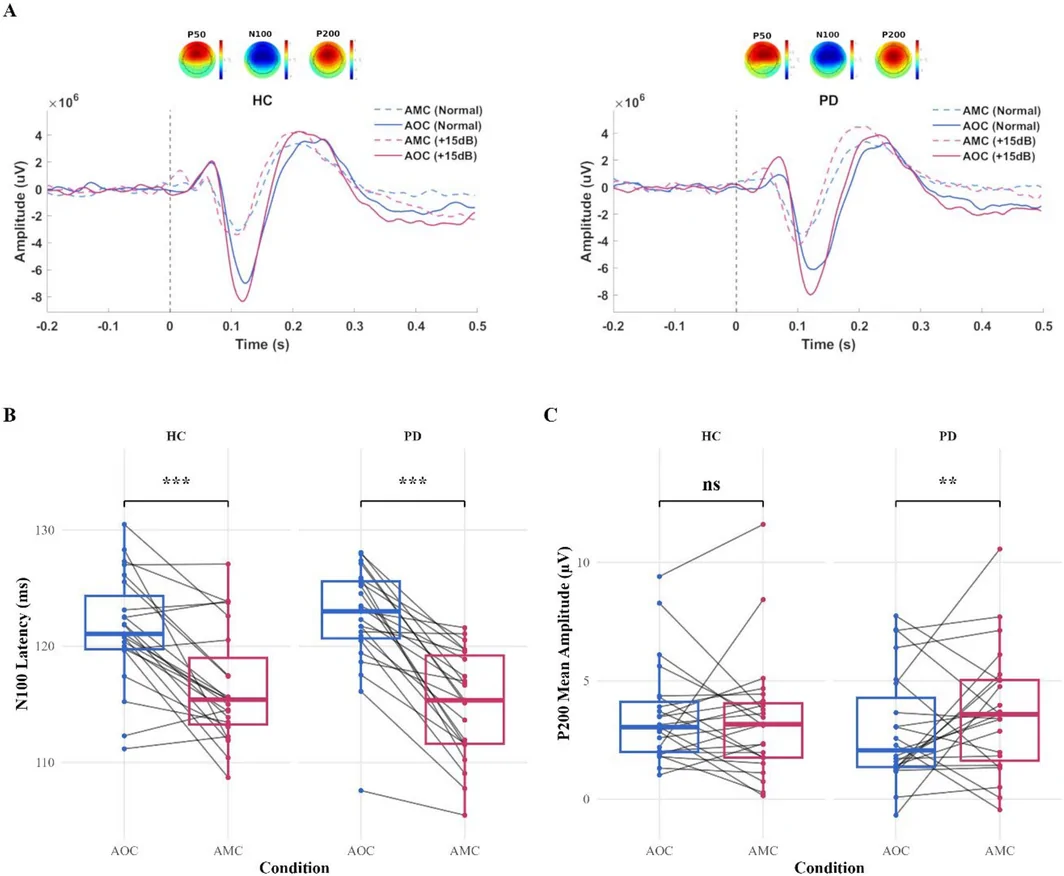
Event‐related potentials (ERPs) and post hoc decomposition of the group × condition interaction. (A) Grand average ERP waveforms and scalp topographies for HC (left) and IwPD (right). ERPs are time‐locked to stimulus onset (vertical dashed line at 0 s) and displayed from −0.2 to 0.5 s. The y‐axis shows amplitude in microvolts (μV). Four conditions were plotted: AMC with normal loudness (dashed blue), AMC with increased loudness (+15 dB; dashed magenta), AOC with normal loudness (solid blue), and AOC with increased loudness (+15 dB; solid magenta). The scalp topographies above each waveform reflect the spatial distribution of the voltage at the peak latency of the P50, N100, and P200 components. Waveforms were averaged over the frontocentral region of interest. (B and C) Post hoc decomposition of the group × condition interaction for (B) N100 latency (ms) and (C) P200 mean amplitude (μV), by condition (AOC vs. AMC) and group (HC and PD). Each black line represents a single participant across conditions; boxplots display group‐level distributions, with dots colored by condition (blue = AOC, magenta = AMC). Asterisks indicate the significance of the AMC‐AOC contrast within each group (***p < 0.001; **p < 0.01; ns, not significant). For N100 latency, both groups showed a significant AMC‐AOC shift, larger in IwPD (Δ = −7.3 ms) than in HC (Δ = −5.1 ms), reflecting a significant group × condition interaction (*p* = 0.012); direct between‐group comparisons within each condition were nonsignificant. For P200 amplitude, the AMC‐AOC difference was significant only in IwPD, likewise reflecting a group × condition interaction (*p* = 0.012).

**TABLE 3:** (Post hoc). Simple‐effects decomposition of group × condition on N100 latency.

Contrast	Estimate	SE	df	t.ratio	*p*
HC: AMC‐AOC	**−0.0051 s**	0.000618	6162	−8.21	***p* < 0.001**
PD: AMC‐AOC	**−0.0073 s**	0.000618	6162	−11.76	***p* < 0.001**
AOC: HC‐PD	−0.0009 s	0.00131	56.2	−0.685	0.496
AMC: HC‐PD	+0.0013 s	0.00131	56.2	0.986	0.329

*Note:* Simple‐effects decomposition of the group × condition interaction on N100 latency. Contrasts show the AMC‐AOC difference within each group and the HC‐PD difference within each condition. Estimates in seconds; significant results in bold. *p* values FDR‐corrected.

#### 3.1.3 P200

For the P200 component, the full model is reported in Table S7 (amplitude) and Table S8 (latency). The group × condition interaction reached significance and was retained for both amplitude models; no three‐way or remaining two‐way interactions survived elimination. The final amplitude model included group, condition, loudness, and group × condition.

A significant main effect of loudness was observed for amplitude (*β* = 0.62, 95% CI [0.12, 1.12], *p* = 0.018), indicating that louder stimuli elicited larger P200 amplitudes. A significant group × condition interaction was also found (*β* = 0.93, 95% CI [0.20, 1.65], *p* = 0.012); no significant effects were found for group or condition alone (see Table 4). The marginal *R* ^2^ was 0.003 and the conditional *R* ^2^ was 0.076 (ICC = 0.073). Post hoc comparisons showed that HC did not differentiate AMC from AOC (estimate = −0.20, *p* = 0.442), whereas IwPD showed a significant amplitude increase for self‐generated relative to passively heard stimuli (estimate = 0.73, *p* = 0.0057). Comparing groups within each condition, direct HC–IwPD differences were nonsignificant (AOC: estimate = 0.67, *p* = 0.309; AMC: estimate = −0.26, *p* = 0.688), indicating that the interaction reflected a group difference in the magnitude of the condition effect rather than an overall amplitude difference between groups at either condition level (see Figure 2 and Table 5).

**TABLE 4:** Effects of group, condition, and loudness on P200 amplitude and latency.

Predictors	Amplitude			Latency		
	Estimates	CI	*p*	Estimates	CI	*p*
Group	−0.66	[−1.91, 0.58]	*p* = 0.300			
Condition	−0.20	[−0.72, 0.31]	*p* = 0.442	**−0.0017**	**[−0.0026, −0.0008]**	***p* < 0.001**
Loudness	**0.62**	**[0.12, 1.12]**	***p* = 0.018**	**−0.0018**	**[−0.0029, −0.0008]**	***p* < 0.001**
Group × condition	**0.93**	**[0.20, 1.65]**	***p* = 0.012**			
**Random effects**	*σ* ^2^ = 53.70 *τ*₀₀ = 3.80 ICC = 0.073 *R* ^2^ = 0.003/0.076			*σ* ^2^ = 3.644e‐4 *τ*₀₀ = 7.0e‐6 *R* ^2^ = 0.004/n.a.		

*Note:* The table shows fixed‐effect estimates, 95% confidence intervals (CIs), and *p* values from mixed‐effects models predicting P200 amplitude and latency. Significant results are shown in bold. Group × condition was retained in the amplitude model; group and group × condition were not retained in the latency model (shaded cells).

**TABLE 5:** (Post hoc). Simple‐effects decomposition of group × condition on P200 amplitude.

Contrast	Estimate	SE	df	t.ratio	*p*
HC: AMC‐AOC	−0.20	0.262	6162	−0.77	*p* = 0.442
PD: AMC‐AOC	**0.73**	0.262	6162	2.77	***p*** **= 0.0057**
AOC: HC‐PD	0.67	0.648	52.6	1.03	*p* = 0.309
AMC: HC‐PD	−0.26	0.648	52.6	−0.40	*p* = 0.688

*Note:* Simple‐effects decomposition of the group × condition interaction on P200 amplitude. Contrasts show the AMC‐AOC difference within each group and the HC‐PD difference within each condition. Estimates in μV; significant results in bold. *p* values FDR‐corrected.

For P200 latency, significant main effects were found for both condition (*β* = −0.0017, 95% CI [−0.0026, −0.0008], *p* < 0.001) and loudness (*β* = −0.0018, 95% CI [−0.0029, −0.0008], *p* < 0.001), reflecting shorter latencies in the AMC condition and with louder stimuli. No significant effect of group was observed, and no interactions survived elimination. The marginal *R* ^2^ was 0.004; the conditional *R* ^2^ could not be estimated (singular fit).

## 4 Discussion

Sensory feedback plays an important role in voice regulation, allowing the central nervous system to monitor and adjust parameters such as loudness, pitch, and articulation in real time (Behroozmand et al. 2016). In IwPD, this regulatory mechanism is likely impaired, contributing to hypophonia and the vocal monotony characteristic of hypokinetic dysarthria (Liu et al. 2012; Huang et al. 2016). One way to investigate the integrity of this feedback system is through EEG, specifically by examining the ERPs elicited during sensory feedback processing in voice tasks. The morphology and amplitude of early auditory ERPs, namely the P50, N100, and P200 components, are thought to reflect the brain's ability to detect and process self‐generated voices. Therefore, altered ERP responses may serve as physiological markers of disrupted sensory feedback in IwPD. The present findings indicate that abnormalities in IwPD emerged specifically in the temporal dynamics of the N100 response and in the amplitude of the P200 component, whereas the earliest stage of sensory processing, indexed by the P50, remained preserved. A plausible interpretation of this pattern of results is that IwPD display altered timing of sensory predictions about their own voice, together with an enhanced late attentional response, particularly for the self‐generated voice.

Modulations in P50 amplitude have been linked to sensory gating, a mechanism that attenuates responses to repeated or predictable stimuli and protects the auditory system from overstimulation (Brinkmann et al. 2021). In our study, P50 was larger in the AOC than in the AMC condition in both groups. Rather than reflecting increased activation to the external stimulus, this pattern suggests an attenuation of the self‐generated voice, in line with the idea that self‐voice production engages sensory consequences of one's own action and reduces the evoked response (Knolle et al. 2019). This attenuation depends on self‐generation rather than acoustic familiarity, since the external voice, although recognizable as one's own, does not show the same suppression. However, no significant group differences emerged in the current study, indicating comparable early sensory responses in PD and HC. This preserved P50 profile contrasts with findings for nonvocal stimuli, including reduced P50 habituation to click stimuli in paired‐stimulus paradigms (De Groote et al. 2020) and increased P50 amplitude responses in tone tasks in early‐stage PD (Hassin‐Baer et al. 2022).

Regarding the P50 latency, both the PD and control groups showed significant differences between AOC and AMC conditions, consistent with the idea that motor preparation facilitates the processing of self‐generated stimuli (Pinheiro et al. 2019). Taken together, the P50 amplitude and latency findings suggest that sensory gating remains intact in IwPD, and that deficits associated with PD may emerge in later ERP components such as N100 and P200 (Hirano et al. 2008).

The N100 component modulation in response to self‐generated sounds reflects auditory cortical suppression, indicating attenuation of expected sensory feedback (Knolle et al. 2013b, 2019). Previous studies have reported reduced or absent N100 suppression effects in IwPD (Huang et al. 2016), suggesting impaired sensory feedback processing in voice pitch‐perturbation paradigms (Contreras‐Ruston et al. 2025). However, here no N100 suppression effect showed at the group level. Instead, a significant condition × loudness interaction was observed, with no differences between IwPD and HC. Specifically, higher‐intensity stimuli produced an increased N100 response in the AOC condition, whereas no changes were observed in the AMC condition. This pattern was similar in both groups. When the stimulus onset was triggered by the participant's own motor action, the N100 was not modulated by stimulus loudness, suggesting that predictions generated by internal models were sufficient to attenuate the influence of acoustic voice loudness. In contrast, during passive listening, neural responses varied proportionally with stimulus loudness, as expected. This is consistent with recent studies showing that self‐generation prevents the N100 amplitude from increasing with stimulus loudness, whereas button‐press–triggered sounds do not produce this effect (Salgado et al. 2026). These findings might suggest that gain modulation of self‐generated stimuli, independent of their loudness, constitutes a general mechanism that is not restricted to voice or to PD. This mechanism could explain the absence of the interaction of loudness across groups and conditions observed in this study.

These results differ from previous reports. Brajot et al. (2016) observed distinct response patterns between IwPD and HC self‐generated voice loudness regulation. Likewise, our findings do not replicate the reduced N100 suppression effect described by Railo et al. (2020), nor the diminished or absent suppression effect reported in other studies (Huang et al. 2016). However, these comparisons should be interpreted with caution, since the results of perturbation paradigms depend largely on the acoustic parameter manipulated in speech production (e.g., pitch), which limits their direct comparability with our sound‐intensity‐based reproduction design. Conversely, the absence of group effects is consistent with the results of previous studies using pitch‐perturbation paradigms, which likewise found no between‐group differences in this measure (Contreras‐Ruston et al. 2025; Huang et al. 2016; Li et al. 2021).

Our results did not confirm altered N100 suppression in IwPD nor group difference. This suggests that early cortical processing of voice loudness remains relatively preserved in IwPD early in the disease progression. This contrasts with electrophysiological evidence that IwPD may exhibit a heightened neural response to the acoustic features of speech: increased frequency‐following response (FFR) amplitude, suggesting enhanced encoding of pitch and loudness at early auditory stages (Mollaei et al. 2022), and increased auditory oscillatory activity in the delta, theta, and alpha bands over parietal regions, associated with altered inhibitory regulation of cortical sensory‐feedback processing (Güdücü et al. 2019). The preserved N100 amplitude in the current IwPD sample may indicate that the behavioral alterations in voice intensity regulation observed in PD (Abur et al. 2021; Clark et al. 2014), particularly those related to sensory feedback, do not originate at processing stages prior to the N100. Instead, these difficulties may emerge at later stages, where sensory predictions are integrated and transformed into motor adaptation of vocal output. Given that our paradigm manipulated the loudness of one's own voice, a central feature of hypophonia, and one frequently underrecognized by IwPD, the absence of group differences in the N100 suppression effect indicates that the origin of these vocal deficits does not lie in the early cortical encoding of voice loudness.

However, group differences showed in the N100 latency. IwPD showed a more pronounced AMC‐AOC latency onset difference than HC. Importantly, this finding does not reflect a general acceleration of auditory processing in PD. Rather, it suggests a specific alteration in the timing how IwPD process self‐generated compared to passively perceived self‐voices, consistent with a forward‐model prediction that is temporally mistuned, possibly generating a temporal mismatch in sensory feedback processing.

This result is consistent with and extends the conclusions of the recent systematic review on vocal production in PD (Contreras‐Ruston et al. 2025), which showed that the N100 amplitude response does not differ between IwPD and HC in pitch‐perturbation paradigms (Huang et al. 2016, 2019; Li et al. 2021; Dai et al. 2022). The review also proposed that manipulating vocal loudness might reveal alterations not detected through pitch changes. Our results partially support this hypothesis, although they suggest temporal rather than amplitude changes. Although this effect did not extend to an interaction at the perceived loudness level, the inclusion of a self‐voice loudness manipulation represents an important extension to previous voice paradigms. In particular, we manipulated the loudness of the speaker's own voice in both active and passive conditions to investigate the difficulties IwPD might experience in adjusting their voice loudness. According to the mechanism proposed by Salgado et al. (2026), self‐generation via a button press could attenuate the auditory response's dependence on stimulus loudness through predictive filtering, which would make the response relatively insensitive to loudness changes. This is consistent with the current.

From the perspective of internal models, this temporal delay suggests that the predictive signal associated with self‐generation is not absent. The suppression effect was present in both groups, but temporally misaligned. As prediction underlies self‐generation, i.e., a motor action precedes sensory consequences, the interaction is best understood as a mistuning in the timing of this motor‐to‐auditory prediction in IwPD, rather than a failure of suppression per se. This misalignment could generate a mismatch between predicted and actual sensory feedback, affecting both its temporal prediction and the dopaminergic weighting of the resulting prediction error (Schwartze and Kotz 2016; Valdés‐Baizabal et al. 2020). This interpretation is consistent with the notion of a more general alteration in auditory temporal prediction mechanisms, rather than a speech‐specific deficit, in line with studies reporting N100 anomalies in nonlinguistic auditory paradigms in aging (Criscuolo et al. 2025). Nonetheless, the contribution of other processes, such as attentional modulation (Praamstra and Oostenveld 2003) or alterations in inhibitory control (Ziri et al. 2024), cannot be ruled out.

In IwPD, the observed pattern was not a diffuse slowing of N100 latency across conditions but a larger AMC‐AOC latency shift than in HC, driven by an earlier AMC peak. This systematic and unidirectional effect, rather than random fluctuation in peak latency, indicates a specific rather than global alteration in temporal processing. This is compatible with the level‐dependent nature of the N100, whose latency is known to track stimulus intensity (Soeta and Nakagawa 2012; see also Uppenkamp and Röhl 2014), such that an exaggerated between‐condition latency change would be more readily detectable in a paradigm like ours.

Within the forward‐model framework, the current evidence remains inconclusive regarding the effect of loudness on the N100, as a study using pure tones of different intensities in IwPD found no significant differences in either N100 amplitude or latency compared with controls (De Keyser et al. 2021). Whether the perception of a self‐generated voice is equivalent to that of a pure tone remains an open question. In this regard, reporting N100 latency effects for self‐voice suppression is indicative of sound complexities affecting latency responses. A pattern of stimulus specificity has also been described in other study contexts than PD. Using a self‐initiation paradigm, Pinheiro et al. (2018) observed N100 alterations for the self‐voice but not for tones in individuals prone to auditory hallucinations. Although this effect was expressed in an N100 amplitude change and in a different population, their findings suggest that self‐voice may constitute a particularly sensitive stimulus for detecting voice‐related changes. P200 amplitude modulations have been associated with auditory–motor integration and compensatory responses to altered sensory feedback (Huang et al. 2019). In PD, an increased P200 amplitude in response to unexpected auditory signals in speech production was observed and interpreted as a heightened corrective effort or cortical compensation when feedforward control is insufficient to stabilize the voice (Liu et al. 2012; Chen et al. 2013; Huang et al. 2016). Feedforward control is thought to be mediated by noncortical structures such as the basal ganglia and cerebellum (Hickok et al. 2011; Tourville and Guenther 2011; Parrell and Houde 2019). Here, the P200 amplitude showed a significant group × condition interaction: IwPD exhibited a larger amplitude for the self‐generated than the passively listened to voice, whereas HC showed no such differentiation. This pattern is consistent with the evidence reviewed by Contreras‐Ruston et al. (2025), in which IwPD showed systematically increased P200 responses during active vocalization relative to both HC and passive listening in pitch‐shift paradigms (Huang et al. 2016; Li et al. 2021; Dai et al. 2022), indicative of greater cognitive demand and allocation of attentional resources to vocal production as a compensatory response to altered feedforward control.

The present findings extend this interpretation to a loudness‐based paradigm. The P200 response appears not specific to pitch perturbations, but may instead reflect a more general mechanism of heightened attentional monitoring of one's own voice in PD, largely independent of the acoustic parameter manipulated. The P200 has been linked to the detection of mismatches between sensory feedback and actual input, saliency detection, and the regulation of attention in sensory feedback (Behroozmand et al. 2009; Duggirala et al. 2023); in this context, the group‐specific increase may reflect a compensatory neural effort in response to the temporal imprecision indexed by the N100, an attempt by the system to resolve the residual sensory discrepancy through the reallocation of attentional resources. This dissociation is also consistent with the idea that the N100 and P200 reflect different stages of auditory processing. Whereas the N100 is related to early sensory encoding, the P200 is associated with a later stage in which the sound and its relevance are evaluated (Knolle et al. 2013a; Salgado et al. 2026), which could also include the sensory feedback of one's own voice. Our results suggest that the alterations in IwPD emerged during this evaluative stage, rather than during the initial encoding of intensity.

The present findings support a reinterpretation of the auditory‐motor deficit in PD: rather than a deficit in sensory feedback processing per se, or in the perception of the externally presented voice, it is the temporal precision of sensory prediction, together with a compensatory attentional response to the self‐voice, that appears to contribute to voice deficits in IwPD. Unlike previous suppression studies, where group differences typically arise from reduced auditory‐motor suppression amplitude, our N100 group effect was expressed in latency rather than amplitude, and was accompanied by a group × condition modulation of the P200. The preserved P50 and the group‐invariant N100 amplitude indicate that early sensory processing stages are intact, and that the most relevant group differences emerge in the temporal dynamics of the N100 and in the amplitude of the P200. Nevertheless, previous studies have reported heterogeneous results regarding early ERP responses in PD, including an increased P200 amplitude interpreted as a compensatory mechanism when the feedforward control system is compromised (Huang et al. 2016; Knolle et al. 2019). Such variability across studies might be attributable to differences in experimental paradigms, measures used (e.g., voice loudness or pitch), and clinical heterogeneity inherent to PD (Contreras‐Ruston et al. 2025).

Altogether, the present study provides novel evidence on voice production and sensory feedback processing in PD. However, some limitations should be noted. First, our relatively small and narrowly defined cohort, 23 IwPD at early‐to‐moderate stages (I–III) under a single medication regimen, plus 23 matched healthy controls, limits the extent to which these findings can be extrapolated to the broader PD population. In particular, IwPD at more advanced stages, on different treatment protocols, of varying ages or cognitive status, may exhibit different early ERP responses. Future work with larger, more diverse samples spanning later disease stages, multiple medication conditions, and broader demographic ranges is needed to confirm the generalizability of the current results. In addition, it was not possible to optimally match participants by age and sex, and the duration of Parkinson's diagnosis varied among the affected individuals. Another limitation relates to the medication status of the participants. All IwPD in this study were assessed in the “ON” state after their usual dopaminergic medication, which differs from studies conducted in the “OFF” state (e.g., Huang et al. 2016). This choice was driven by both clinical and methodological considerations: Testing IwPD OFF medication would have increased motor symptoms, discomfort, and fatigue during relatively long EEG recordings, and, crucially, would have exacerbated movement‐related artefacts. Assessing participants in their habitual ON state is therefore common practice in EEG and speech PD studies (Sörös et al. 2017; Dai et al. 2022), as it improves tolerability and data quality. From a mechanistic perspective, in the absence of medication, low dopamine levels can be associated with inadequate filtering of unexpected stimuli, leading to sensory dysregulation and a discrepancy between the actual and perceived voice, favoring hypophonia and reduced voice awareness. With levodopa, this filtering mechanism can be partially restored, improving the temporal coordination of auditory‐motor integration, though incompletely, as dopaminergic treatment has clearer effects on putaminal motor circuits than on mesencephalic auditory pathways (Valdés‐Baizabal et al. 2020). Given the established role of basal‐ganglia circuits in temporal processing (Schwartze and Kotz 2016), this framework is compatible with our central finding of a AMC‐AOC latency shift in the ON state, which may reflect a residual imprecision in the timing of sensory prediction that dopaminergic treatment does not fully normalize. However, results on how medication affects sensory processing in PD are inconsistent; some studies report improvements, while others find no differences, likely depending on the type of stimulus involved (Georgiev et al. 2015). Furthermore, regarding voice parameters, a systematic review indicated that dopaminergic status influences fundamental frequency but not intensity, suggesting that the effects on loudness regulation remain limited (Pinho et al. 2018). This aligns with the group‐invariant, loudness‐dependent N100 amplitude modulation observed here. Future studies that systematically compare ON and OFF states in the same individuals will therefore be essential to determine to what extent the present ON‐state effects are directly dopamine‐dependent.

Finally, our paradigm was not a speech or voice production task in the usual sense. The button press generates an efference copy of a voluntary action, but it does not carry the articulatory commands that would predict the sensory consequences of voice loudness. Any relevance of these findings to speech production therefore rests on a further assumption, that self‐generated sensorimotor prediction in PD is not effector‐specific, and remains a hypothesis for future work (Puyjarinet et al. 2019). Accordingly, any attenuation observed here may reflect general predictive mechanisms of self‐generated action rather than a mechanism specific to voice loudness. The paradigm also used isolated sustained vowels and thus lacks the communicative richness of spontaneous speech or reading aloud. Nonetheless, dissociating self‐voice processing from overt articulation is also a strength, as it minimizes articulatory, somatosensory, and muscle‐related confounds (including EEG artefacts) and provides a cleaner measure of the predictive auditory processing associated with self‐generated actions. Although motor‐related activity differences between groups were controlled for by the individual‐level MOC subtraction, future research directly comparing motor activity and auditory feedback processing in IwPD could provide a more comprehensive characterization of the motor‐to‐auditory interactions underlying self‐voice monitoring deficits in this population. Indeed, previous work in healthy participants using similar playback designs has demonstrated robust N100 suppression for self‐initiated versus externally generated sounds (Pinheiro et al. 2019, 2020).

In IwPD, this suppression was likewise present, but temporally altered, with an exaggerated AMC‐AOC latency shift. Building on this, future studies could manipulate the delay between the motor action and playback in comparable self‐initiated paradigms (Korzyukov et al. 2017), incorporate more naturalistic stimuli such as words or phrases to approximate spontaneous speech, and contrast the self‐voice with other self‐generated sounds to establish whether the observed effects are specific to one's own voice or reflect a more general sensitivity to self‐generated input.

Despite these limitations, these results encourage future research on the role of sensory feedback processing in PD speech production. We consider that this approach could be further complemented by neuroimaging methods. Because our N100 group effect concerns the timing of auditory‐motor prediction, EEG‐based connectivity measures such as coherence and phase‐locking would be particularly informative, as they can test whether motor and auditory regions synchronize appropriately during self‐voice processing, something single‐electrode amplitude or latency cannot capture. Furthermore, the implementation of paradigms that combine speech‐induced suppression with motor‐related potentials, such as priming potentials (Tremblay and Sato 2024), could provide a complementary characterization of how motor priming and auditory feedback interact during speech production in the IwPD. Responses occurring after 300 ms, reflecting the evaluation and updating of internal models following unexpected auditory feedback, reveal how IwPD detect and integrate mismatches in their self‐generated voice. This idea is supported by Suchý et al. (2023), who demonstrated in healthy controls that conscious detection of pitch‐shifted feedback errors elicits a robust late positive ERP (500–700 ms), correlating with compensatory vocal responses and involving temporal, frontal, and parietal networks.

From a clinical perspective, the current findings highlight the need to develop tools that enable visualization and quantification of voice self‐perception in IwPD, such as real‐time auditory feedback interfaces. Furthermore, both auditory assessments (Castillo‐Allendes et al. 2021) and voice therapies or speech technologies targeting this population should explicitly incorporate training strategies that address sensory feedback. Focusing on these sensory processes could enhance the regulation of vocal loudness and, in turn, improve everyday communication in IwPD.

## 5 Conclusions

This study set out to understand voice production and its deficits in PD, but the paradigm we used measured the neural processing of a self‐generated voice, not speech or voice production per se. Conclusions are therefore confined to the former, and any bridge to the latter is offered as an explicit conjecture. IwPD displayed altered temporal dynamics in the processing of their own voice, rather than hypersensitivity to loudness changes. The earliest sensory stage, indexed by the P50, and the intensity‐dependent modulation of N100 amplitude were preserved and comparable to controls. These findings indicate that early cortical encoding of self‐voice loudness remains intact. Instead, the group difference emerged in N100 latency; IwPD showed an exaggerated temporal separation between the AMC and AOC, accompanied by an enhanced P200 response to their self‐generated voice. This pattern points to altered temporal rather than absent or reduced cortical processing of one's own voice, together with a compensatory increase in attentional monitoring. Thus, the current findings reveal a deficit in the temporal precision of auditory self‐voice monitoring; whether this dysfunction extends to other auditory inputs remains to be determined.

Whether this temporal imprecision relates to reduced voice loudness characteristic of PD cannot be established here: our results speak to self‐voice processing rather than to voice production, and any association would require that self‐generated sensorimotor prediction in PD is not effector specific. This is a hypothesis for future work that directly combines production and perception measures. From the clinical point of view, to the extent that auditory self‐monitoring proves relevant to vocal output, this would underscore the importance of incorporating rehabilitation strategies that strengthen auditory feedback processing and recalibrate how IwPD perceive their own voice, with the aim of supporting vocal loudness regulation and mitigating the communicative impact of hypophonia in these individuals.

## Author Contributions

**Francisco Contreras‐Ruston:** conceptualization, data curation, formal analysis, methodology, visualization, writing – original draft, writing – review and editing. **Antonio Criscuolo:** data curation, formal analysis, visualization, writing – review and editing. **Antonio Callén:** investigation, writing – review and editing. **David Cucurell:** resources, software. **Jordi Navarra:** conceptualization, methodology, formal analysis, writing – original draft, writing – review and editing, supervision. **Sonja A. Kotz:** conceptualization, methodology, formal analysis, writing – original draft, writing – review editing, supervision.

## Funding

This study was supported by Agencia Chilena de Investigación y Desarrollo (ANID) (Beca Chile para Estudios en el Extranjero 2020, folio 72210019) and the Generalitat de Catalunya (2021 SGR 00352), and funded by a Lundbeck Foundation postdoctoral grant (ref R521‐2025‐1777).

## Conflicts of Interest

The authors declare no conflicts of interest.

## Supporting information


**Table S1:** Full model (Type III ANOVA)—P50 amplitude.

**Table S2:** Full model (Type III ANOVA)—P50 latency.

**Table S3:** Effects of condition on P50 amplitude and group, condition on P50 latency.

**Table S4:** Full model (Type III ANOVA)—N100 amplitude.

**Table S5:** (Post hoc). Simple‐effects decomposition of condition × loudness on N100 amplitude.

**Table S6:** Full model (Type III ANOVA) — N100 latency.

**Table S7:** Full model (Type III ANOVA)—P200 amplitude.

**Table S8:** Full model (Type III ANOVA)—P200 latency.

## Data Availability

Due to ethical restrictions related to patient confidentiality, raw data cannot be shared publicly. However, the full analysis code will be made openly accessible on GitHub.
