# Supplementary material for: Altered Timing of Self‐Voice Processing in Parkinson's Disease
Source: Eur J Neurosci. 2026 Oct 14;64(7):e70700. doi: 10.1111/ejn.70700 (PMC13669414; doi:10.1111/ejn.70700)
Supplement: Supplementary file 1 [file EJN-64-0-s001.docx]

**Table S2. Full Model (Type III ANOVA) - P50 Latency**

| **Term** | **F** | **df** | **p** |
| --- | --- | --- | --- |
| Group | **4.33** | 1, 22.1 | **p=0.049** |
| Condition | **53.69** | 1, 6172.6 | **p<0.001** |
| Loudness | 0.98 | 1, 50.3 | p=0.328 |
| Group × Condition | 2.74 | 1, 6172.6 | p=0.098 |
| Group × Loudness | 0.07 | 1, 50.3 | p=0.793 |
| Condition × Loudness | 2.60 | 1, 6172.6 | p=0.107 |
| Group × Condition × Loudness | 1.46 | 1, 6172.6 | p=0.227 |

*Note:* Full model (Type III ANOVA with Satterthwaite's method) for P50 latency, showing all main effects and interactions before backward elimination. F-values, degrees of freedom (numerator, denominator), and p-values are reported for each term. Significant effects are shown in bold. Group = HC vs. IwPD; Condition = AOC vs. AMC; Loudness = Normal vs. +15 dB.
