# Supplementary material for: Altered Timing of Self‐Voice Processing in Parkinson's Disease
Source: Eur J Neurosci. 2026 Oct 14;64(7):e70700. doi: 10.1111/ejn.70700 (PMC13669414; doi:10.1111/ejn.70700)
Supplement: Supplementary file 2 [file EJN-64-0-s002.docx]

**Table S7. Full Model (Type III ANOVA) - P200 Amplitude**

| **Term** | **F** | **df** | **p** |
| --- | --- | --- | --- |
| Group | 0.05 | 1, 46 | p=0.818 |
| Condition | 1.99 | 1, 6164 | p=0.158 |
| Loudness | **6.21** | 1, 46 | **p=0.016** |
| Group × Condition | **6.25** | 1, 6164 | **p=0.012** |
| Group × Loudness | 0.65 | 1, 46 | p=0.424 |
| Condition × Loudness | 0.32 | 1, 6164 | p=0.574 |
| Group × Condition × Loudness | 0.17 | 1, 6164 | p=0.679 |

*Note:* Full model (Type III ANOVA with Satterthwaite's method) for P200 amplitude, showing all main effects and interactions before backward elimination. F-values, degrees of freedom (numerator, denominator), and p-values are reported for each term. Significant effects are shown in bold. Group = HC vs. IwPD; Condition = AOC vs. AMC; Loudness = Normal vs. +15 dB.
