# Supplementary material for: Altered Timing of Self‐Voice Processing in Parkinson's Disease
Source: Eur J Neurosci. 2026 Oct 14;64(7):e70700. doi: 10.1111/ejn.70700 (PMC13669414; doi:10.1111/ejn.70700)
Supplement: Supplementary file 3 [file EJN-64-0-s003.docx]

**Table S5 (Post-hoc). Simple-Effects Decomposition of Condition × Loudness on N100 Amplitude**

| **Contrast** | **Estimate** | **SE** | **df** | **t.ratio** | **p** |
| --- | --- | --- | --- | --- | --- |
| AOC: 15dB − Normal | **-1.09** | 0.307 | 99.8 | -3.54 | **p<0.001** |
| AMC: 15dB − Normal | 0.22 | 0.307 | 99.8 | 0.72 | p=0.472 |
| Normal: AMC − AOC | **2.75** | 0.249 | 6162 | 11.03 | **p<0.001** |
| 15dB: AMC − AOC | **4.06** | 0.249 | 6162 | 16.28 | **p<0.001** |

*Note:* Simple-effects decomposition of the Condition × Loudness interaction on N100 amplitude (µV). Contrasts show the 15 dB − Normal difference within each condition (AOC, AMC) and the AMC − AOC difference within each loudness level (Normal, +15 dB). Estimates in µV; significant results in bold. p-values FDR-corrected. AOC = auditory-only condition; AMC = auditory-motor condition.
