# Supplementary material for: Altered Timing of Self‐Voice Processing in Parkinson's Disease
Source: Eur J Neurosci. 2026 Oct 14;64(7):e70700. doi: 10.1111/ejn.70700 (PMC13669414; doi:10.1111/ejn.70700)
Supplement: Supplementary file 4 [file EJN-64-0-s004.docx]

## **Table S3.** Effects of Condition on P50 Amplitude and Group, Condition on P50 Latency

| **Predictors** | **Amplitude** | | | **Latency** | | |
| --- | --- | --- | --- | --- | --- | --- |
|  | **Estimates** | **CI** | **p** | **Estimates** | **CI** | **p** |
| Group |  |  |  | -0.0007 | [-0.0013, -0.0001] | p=0.093 |
| Condition | **-0.88** | **[-1.23, -0.53]** | **p<0.001** | **-0.0022** | **[-0.0028, -0.0016]** | **p<0.001** |
| Loudness |  |  |  |  |  |  |
| **Random** **Effects** | σ² = 50.63 τ₀₀ ≈ 0 R² = 0.004 / n.a. | | | σ² = 1.384e-4 τ₀₀ ≈ 0 R² = 0.009 / n.a. | | |

Note: No Group-related effect was retained in either final model. Group and Loudness were not retained in the amplitude model; Loudness was not retained in the latency model (shaded cells). Full model ANOVA in Supplementary Tables S2–S3 below.
