# Supplementary material for: Altered Timing of Self‐Voice Processing in Parkinson's Disease
Source: Eur J Neurosci. 2026 Oct 14;64(7):e70700. doi: 10.1111/ejn.70700 (PMC13669414; doi:10.1111/ejn.70700)
Supplement: Supplementary file 5 [file EJN-64-0-s005.docx]

**Table S4. Full Model (Type III ANOVA) - N100 Amplitude**

| **Term** | **F** | **df** | **p** |
| --- | --- | --- | --- |
| Group | 0.01 | 1, 46 | p=0.906 |
| Condition | **373.37** | 1, 6164 | **p<0.001** |
| Loudness | 3.02 | 1, 46 | p=0.089 |
| Group × Condition | 2.79 | 1, 6164 | p=0.095 |
| Group × Loudness | 0.05 | 1, 46 | p=0.828 |
| Condition × Loudness | **13.80** | 1, 6164 | **p<0.001** |
| Group × Condition × Loudness | 1.30 | 1, 6164 | p=0.254 |

*Note:* Full model (Type III ANOVA with Satterthwaite's method) for N100 amplitude, showing all main effects and interactions before backward elimination. F-values, degrees of freedom (numerator, denominator), and p-values are reported for each term. Significant effects are shown in bold. Group = HC vs. IwPD; Condition = AOC vs. AMC; Loudness = Normal vs. +15 dB.
