# Supplementary material for: Altered Timing of Self‐Voice Processing in Parkinson's Disease
Source: Eur J Neurosci. 2026 Oct 14;64(7):e70700. doi: 10.1111/ejn.70700 (PMC13669414; doi:10.1111/ejn.70700)
Supplement: Supplementary file 6 [file EJN-64-0-s006.docx]

**Table S6. Full Model (Type III ANOVA) — N100 Latency**

| **Term** | **F** | **df** | **p** |
| --- | --- | --- | --- |
| Group | 0.04 | 1, 46 | p=0.846 |
| Condition | **199.37** | 1, 6210 | **p<0.001** |
| Loudness | **29.76** | 1, 3526.2 | **p<0.001** |
| Group × Condition | **6.30** | 1, 6210 | **p=0.012** |
| Group × Loudness | 0.37 | 1, 3526.2 | p=0.545 |
| Condition × Loudness | 0.62 | 1, 6210 | p=0.431 |
| Group × Condition × Loudness | 0.01 | 1, 6210 | p=0.932 |

Note: Full model (Type III ANOVA with Satterthwaite's method) for N100 latency, showing all main effects and interactions before backward elimination. F-values, degrees of freedom (numerator, denominator), and p-values are reported for each term. Significant effects are shown in bold. Group = HC vs. IwPD; Condition = AOC vs. AMC; Loudness = Normal vs. +15 dB.
