# Supplementary material for: Altered Timing of Self‐Voice Processing in Parkinson's Disease
Source: Eur J Neurosci. 2026 Oct 14;64(7):e70700. doi: 10.1111/ejn.70700 (PMC13669414; doi:10.1111/ejn.70700)
Supplement: Supplementary file 7 [file EJN-64-0-s007.docx]

**Table S1. Full Model (Type III ANOVA) - P50 Amplitude**

| **Term** | **F** | **df** | **p** |
| --- | --- | --- | --- |
| Group | 0.03 | 1, 29.9 | p=0.856 |
| Condition | **24.02** | 1, 6191.5 | **p<0.001** |
| Loudness | 3.29 | 1, 48.5 | p=0.076 |
| Group × Condition | 0.90 | 1, 6191.5 | p=0.343 |
| Group × Loudness | 1.10 | 1, 48.5 | p=0.300 |
| Condition × Loudness | 3.24 | 1, 6191.5 | p=0.072 |
| Group × Condition × Loudness | 0.11 | 1, 6191.5 | p=0.735 |

*Note:* Full model (Type III ANOVA with Satterthwaite's method) for P50 amplitude, showing all main effects and interactions before backward elimination. F-values, degrees of freedom (numerator, denominator), and p-values are reported for each term. Significant effects are shown in bold. Group = HC vs. IwPD; Condition = AOC vs. AMC; Loudness = Normal vs. +15 dB.
